# Peptidoglycan Remodeling in Gram-Negative Bacteria: From Stress Adaptation to Antibiotic Tolerance and Therapeutic Targeting

**DOI:** 10.3390/antibiotics15080815

**Published:** 2026-08-21

**Authors:** Theresa Strohhammer, Alessandra M. Martorana, Alessandra Polissi

**Affiliations:** Department of Pharmacological and Biomolecular Sciences “Rodolfo Paoletti”, University of Milano, 20133 Milano, Italy; theresa.strohhammer@unimi.it (T.S.); alessandra.martorana@unimi.it (A.M.M.)

**Keywords:** PG remodeling, gram-negative bacteria envelope stress, antibiotic tolerance

## Abstract

The peptidoglycan (PG) sacculus of Gram-negative bacteria is continuously reorganized by a diverse enzymatic repertoire, including lytic transglycosylases, endopeptidases, carboxypeptidases, amidases and LD-transpeptidases, that operate alongside PG synthases to maintain envelope integrity throughout the cell cycle. This remodeling machinery has been extensively characterized in the context of growth and division and it is now emerging also as a key determinant of bacterial survival under non-growing and stress conditions. This review summarizes current knowledge on PG remodeling in Gram-negative bacteria, with emphasis on its regulation during stationary phase, environmental stress, and outer membrane perturbation. How these remodeling pathways, characterized by increased 3–3 cross-linking and enhanced PG–outer membrane coupling, contribute to survival under β-lactam exposure and how related enzymatic configurations can give rise to antibiotic tolerance and resistance is also discussed. Finally, recent progress in targeting PG remodeling enzymes, particularly lytic transglycosylases and peptidases, as adjuvant strategies to potentiate existing antibiotics are reviewed. In summary, PG remodeling represents a mechanistically validated but still underexploited target for addressing antibiotic tolerance and resistance in Gram-negative pathogens.

## 1. Introduction

The clinical success of antibiotics that target peptidoglycan (PG) synthesis has for decades been worsened by an underestimated problem: bacterial survival does not necessarily require genetic resistance, but can instead arise from a physiological, often transient, capacity to tolerate antibiotic exposure [1]. This phenomenon reflects the existence of cellular adaptation programs that allow bacterial cells to remain viable without growing, only to resume proliferation once the selective pressure is removed. In this context, PG remodeling emerges as a central homeostatic process [2]. The PG sacculus, that contributes to sustaining the Gram-negative cell integrity, is continuously reorganized by a diverse enzymatic repertoire whose activity is spatially and temporally coordinated by multiprotein complexes such as the elongasome and divisome [3]. This dynamic balance between synthesis and hydrolysis supports not only growth and division but also constitutes the primary mechanism by which Gram-negative bacteria respond to growth arrest, environmental stresses and outer membrane (OM) perturbations, generating specific enzymatic configurations that frequently converge on alternative PG cross-linking modes and increase in OM to PG connection. Indeed, the same enzymatic machineries, that maintain envelope homeostasis, are involved in responses to several conditions that the cell exploits, giving the basis to develop tolerance, stress adaptation, and, in some cases, antibiotic resistance. Despite this mechanistic importance, no antibiotic currently in clinical use directly targets PG remodeling enzymes, which represent a gap of unexploited opportunities. Therefore, this review examines the PG remodeling in Gram-negative bacteria, its regulation across diverse stress and growth-arrest conditions, its role in β-lactam tolerance and resistance, and the therapeutic exploitation of these pathways.

## 2. Peptidoglycan Architecture and Synthesis

**Figure 1:**
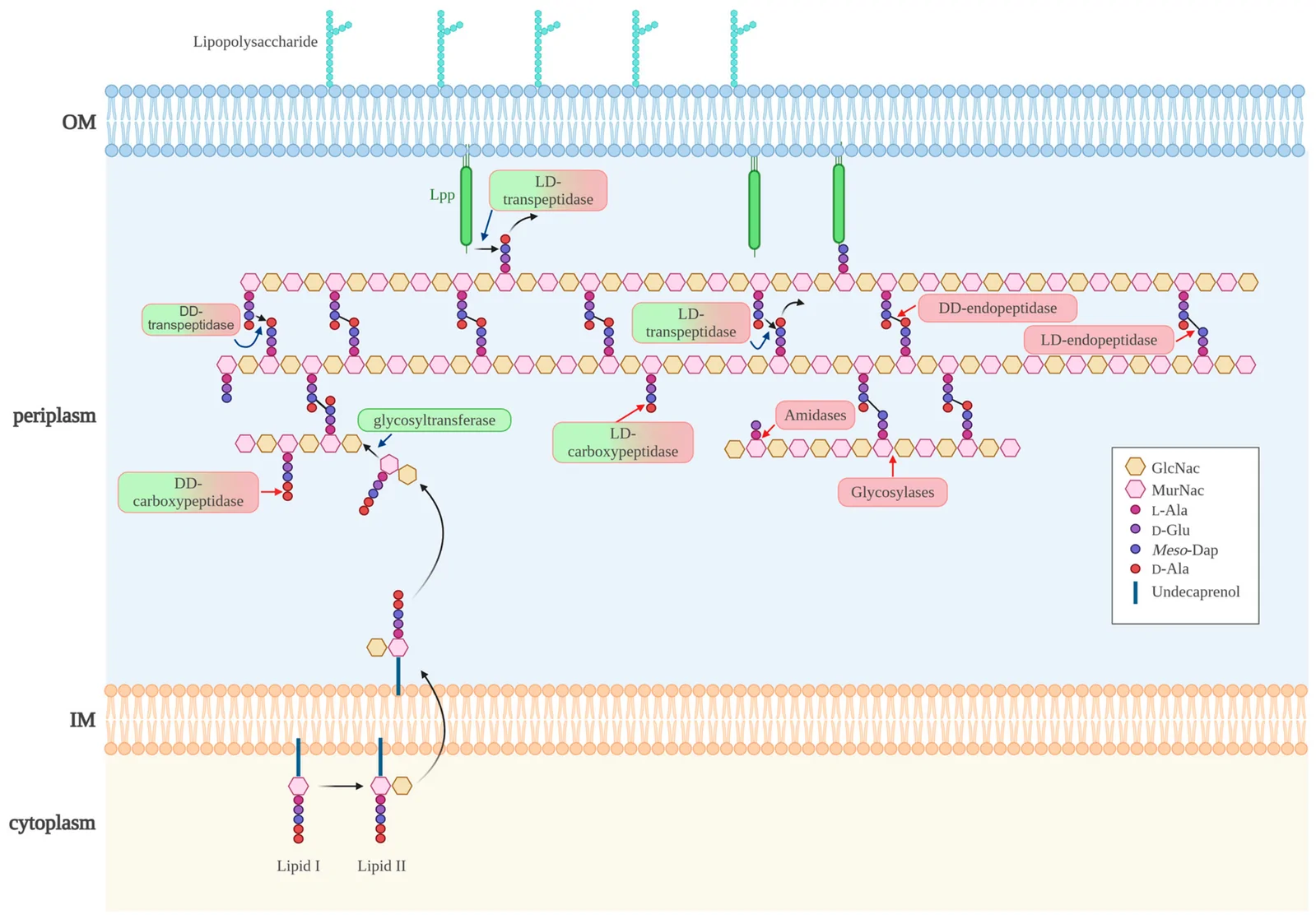
Schematic depiction of the Gram-negative bacterial cell wall, highlighting peptidoglycan synthesis and remodeling proteins. The diagram is organized into four layers, from top to bottom: the outer membrane (OM), which contains lipopolysaccharides (LPS); the periplasm, where the peptidoglycan (PG) resides; the inner membrane (IM); and the cytoplasm. The PG layer is covalently connected to the OM through Braun’s lipoprotein (Lpp). The PG biosynthesis process begins in the cytoplasm, where precursor molecule Lipid I is converted into Lipid II. These precursors consist of a sugar unit (a combination of GlcNAc and MurNAc) attached to a short peptide chain made up of L-alanine, D-glutamate, meso-diaminopimelic acid, and two D-alanine residues, all anchored to the inner leaflet of the IM by the lipid carrier undecaprenol. Once Lipid II is assembled, it is flipped across the IM into the periplasm. For simplicity, the enzymatic steps underlying Lipid I/Lipid II assembly and the mechanism of Lipid II flipping across the IM are not depicted in molecular detail. In the periplasm, an enzyme called glycosyltransferase links the sugar-peptide units to form long glycan strands. These strands are then cross-linked to one another by DD-transpeptidases and LD-transpeptidases, which form peptide bonds between adjacent strands and give the cell wall its mesh-like strength. At the same time, other enzymes work to trim and remodel this structure: DD-carboxypeptidases and LD-carboxypeptidases remove the terminal D-alanine from the peptide stems, while DD-endopeptidases and LD-endopeptidases cut existing cross-links to allow the wall to be reshaped or expanded. Amidases cleave the bonds between the sugar backbone and the peptide stem, and glycosylases cleave within GlcNAc and MurNAc, both of which help the cell turn over old material or insert new strands. Lpp is attached to the PG by LD-transpeptidase activity. Throughout the figure, enzyme labels are color-coded to indicate their functional role: green boxes denote enzymes involved in PG biosynthesis, while red boxes denote enzymes involved in PG remodeling or turnover. Where a label is shown with both colors, this indicates that the enzyme contributes to both biosynthetic and remodeling functions. Blue arrows indicate synthetic (biosynthesis) reactions, while red arrows indicate hydrolytic (degradative) reactions. Created in BioRender. Strohhammer, T. (2026), https://BioRender.com/26rqdz2 (accessed on 31 July 2026).

The cell envelope of Gram-negative bacteria consists of two membranes, the outer (OM) and inner (IM) membrane, which define an aqueous region, the periplasm, harboring the PG [4]. The PG forms a net-like sacculus stretched over the whole cell and its synthesis requires strict coordination of the functions which shape the cell envelope during cell growth and division. It is composed of N-acetylglucosamine (GlcNAc) and N-acetylmuramic acid (MurNAc) disaccharides, cross-linked by β-1,4 glycosidic bonds, with stem peptides attached to MurNAc moieties (Figure 1) [5,6]. In *Escherichia coli*, the pentapeptide chain in nascent PG typically consists of L-Ala, D-Glu, meso-diaminopimelic acid (*m*-Dap), and two D-Ala residues whereof the last D-Ala residue is lost in mature PG [7,8]. Cross-linking occurs primarily between the peptide’s carboxyl group of the D-Ala at fourth position and the ε-amino group of *m*-Dap at third position of another peptide, generating the characteristic mesh-like architecture. In *E*. *coli*, these so-called DD- or 4–3 cross-links are the vast majority, while a smaller fraction (about 2–16%) consists LD- or 3–3 cross-links (between the carboxyl group and the amino group of the *m*-Dap in position three of two adjacent peptides), with the proportions varying according to growth conditions [9]. Braun’s lipoproteins (Lpp) are the most abundant lipoproteins in *E. coli*, they anchor the periplasm facing side of the OM to the PG by being covalently bound to it as a trimeric α-helical coil [10,11]. Unlike Lpp, the OM protein OmpA and the OM Pal lipoprotein, a component of the transenvelope Tol-Pal system, anchor the OM to PG through non-covalent interactions, providing flexible mechanical connections that preserve envelope integrity [12,13].

The PG biosynthetic pathway in Gram-negative bacteria starts in the cytoplasm with the formation of nucleotide-activated sugar precursors (UDP-N-acetylglucosamine and UDP-N-acetylmuramyl pentapeptide) [14]. At the inner leaflet of the IM the precursors are equipped with the lipid carrier undecaprenyl phosphate and form a disaccharide pentapeptide monomer (lipid II) [15,16]. These PG subunits are subsequently flipped across the IM and incorporated into the growing PG matrix in the periplasm. There, PG synthases, including SEDS (Shape, Elongation, Division and Sporulation) proteins and penicillin-binding proteins (PBPs), mediate transglycosylation and transpeptidation, leading to the formation of a cross-linked sacculus (Figure 1) [17,18]. In *E. coli* class A PBPs (PBP1a, PBP1b and PBP1c) are bifunctional (with transglycosylase and transpeptidase activity), whereas class B (PBP2 and PBP3) only have a C-terminal transpeptidase domain. PBPs with a transpeptidase domain catalyze 4-3 cross-links, whereas 3–3 cross-links are catalyzed by LD-transpeptidases (LDTs). LDTs belong to the YkuD-domain protein family and are characterized by active-site cysteine residues, in contrast to PBPs, which generally use an active-site serine [19]. In *E. coli* LdtD and LdtE are LDTs introducing 3–3 cross-links in the PG [19,20], whereas LdtA, LdtB and LdtC, catalyze the formation of the covalent peptide bond between the ε-amino group of the Lpp terminal lysine (Lys 58) and the α-carboxyl group of *m*-Dap in the PG peptide chain, anchoring Lpp to PG [10,11,21]. Finally, DpaA (LdtF) is an atypical LDT family member, that misses a conserved arginine in its catalytic YkuD domain. DpaA hydrolyzes the peptide bond between Lpp terminal Lys and *m*-Dap thus detaching Lpp from PG [22].

PG cannot be considered a static, rigid structure but instead undergoes continuous reorganization to accommodate cell growth, division, and environmental adaptation. These dynamic processes are spatially and temporally coordinated by two major multiprotein complexes associated with the IM: the elongasome and the divisome [3]. The elongasome (Rod system) directs lateral PG insertion during cell elongation, is guided by the actin-like protein MreB and controls the insertion of new PG material along the lateral wall thanks to the combined action of RodA (SEDS protein) and PBP2, thereby maintaining its rod shape [23]. The divisome, whose major components are FtsW (SEDS protein), PBP3 and PBP1b [3], orchestrates septal PG synthesis and cytokinesis and is organized around the tubulin-like protein FtsZ, which forms the Z-ring at mid cell and coordinates septal PG synthesis and constriction [24]. In both complexes the integration of PG synthases, hydrolases, cytoskeletal elements, and regulatory factors, ensure precise spatial and temporal control of PG assembly throughout the cell cycle [3].

## 3. Peptidoglycan Remodeling During Cell Elongation and Division

Along with PG synthesis, remodeling is driven by a diverse set of periplasmic hydrolases that mediate the controlled cleavage of existing PG bonds. During cell elongation and division, the synthesis of PG is intimately connected with its hydrolysis, and the perturbation of this equilibrium compromises cell envelope integrity and viability. Therefore, the elongasome and divisome complexes comprise PG synthase, their regulators and PG hydrolases essential for breaking bonds within the wall to accommodate the insertion of new material [25]. PG hydrolases are essential not only for enabling the insertion of newly synthesized material but also for maintaining sacculus architecture and facilitating adaptation to environmental changes [26]. Maintaining the balance between PG synthesis and hydrolysis is vital for bacterial growth, shape, and viability, highlighting these processes and their coordination as attractive targets for antibiotic intervention [27,28]. Since PG’s main role is to allow cells to withstand turgor pressure generated by osmotic differences between the cell and the environment, PG imbalance can dramatically alter the cell’s fitness and might eventually lead to cell lysis [7]. This chapter will shortly describe the most important PG remodeling factors that contribute to the maintenance of PG homeostasis during growth and division in Gram-negative bacteria.

PG glycosylases cleave within the glycan strands of PG and fall into three groups based on their substrate specificity and catalytic mechanism (Figure 1). The first two, glucosaminidases and muramidases, are hydrolytic enzymes that cleave the *β*-1,4-glycosidic bond between GlcNAc and MurNAc [29]. The third group, lytic transglycosylases (LTGs) such as Slt and the Mlt enzymes in *E. coli*, instead cleave the bond between MurNAc and GlcNAc non-hydrolytically, generating 1,6-anhydroMurNAc residues. Together, all three classes contribute to PG turnover during cell wall expansion, recycling, and quality control of the sacculus, whereof LTGs dominate the periplasmic glycosidase PG remodeling activity [7,8,29]. Endopeptidases play a central role in PG expansion by cleaving peptide cross-links, thereby generating space for the incorporation of new glycan strands and are classified by substrate specificity (Figure 1). These include LD-endopeptidases which target 3–3 cross-links and DD-endopeptidases which target 4–3 cross-links; the latter are β-lactam-insensitive [30,31,32]. Carboxypeptidases modulate the number of cross-links by removing terminal residues from stem peptides in the PG and can also be divided into DD- and LD-carboxypeptidases. Among DD-carboxypeptidases in *E. coli*, there are class C PBPs (PBP4, PBP4b, PBP5, PBP6a and PBP6b), which cleave the terminal D-Ala from pentapeptides, whereas LD-carboxypeptidases cleave tetrapeptides. PBP4 is not only a DD-carboxypeptidase but also has DD-endopeptidase activity [33,34,35] (Table 1). The activity of these enzymes is tightly regulated and coordinated with PG synthesis to ensure proper cell growth. During cell division FtsW and PBP3 form the major PG synthase complex that promote septum formation and PG hydrolases are needed for splitting the septal PG for the separation of the daughter cells [27]. Amidases, AmiA, AmiB and AmiC in *E. coli* remove the stem peptides from the glycan strands producing “denuded” strands and their activity is tightly regulated by LytM domain containing activators (Figure 1) [36,37]. SPOR-domain (SPOrulation-related Repeat domains) proteins bind “denuded” glycan strand and localize to sites of septal PG processing where they coordinate PG remodeling in the final stages of cytokinesis [37,38]. In addition, periplasmic proteases modulate the abundance and activity of both hydrolases and synthases, thereby preventing excessive PG degradation and ensuring proper cell wall assembly [39,40,41,42].

The relative contribution of these remodeling events changes dramatically depending on the physiological state of the cell. During exponential growth they primarily support cell wall expansion, whereas under growth arrest or under stress they become essential maintenance processes that preserve envelope integrity in the absence of substantial PG synthesis.

## 4. Peptidoglycan Remodeling in Non-Growing Conditions

**Figure 2:**
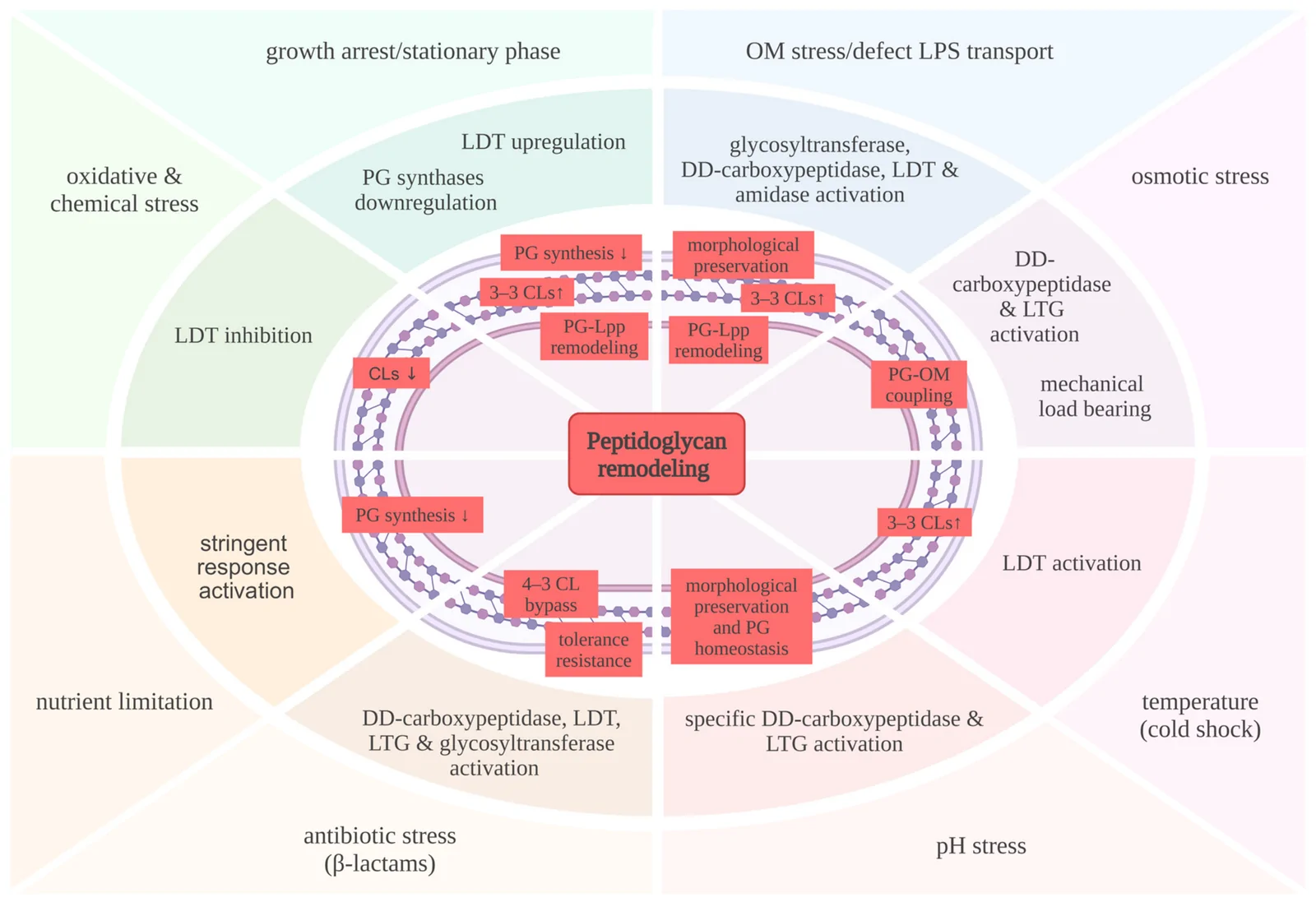
Peptidoglycan remodeling pathways during diverse envelope and physiological stresses in Gram-negative bacteria. Schematic overview of stress-specific peptidoglycan (PG) remodeling responses discussed in this review. The outer ring indicates the stress condition to which the cell must adapt; the middle ring indicates the specific remodeling pathway, enzyme class, or regulatory system engaged (activated or inhibited) in response to that stress; the inner ring indicates the resulting structural or functional outcome for the PG, arrows indicate increased or decreased abundance of the corresponding PG remodeling. Starting top left, going clockwise: During growth arrest or stationary phase, LDTs are upregulated while PG synthases are downregulated, decreasing PG synthesis, increasing 3–3 cross-links (CLs), and driving PG–Lpp remodeling. Under OM stress or defective LPS transport, glycosyltransferases, DD-carboxypeptidases, LDTs and amidases are activated, preserving cell morphology, increasing 3–3 CLs and engaging in PG–Lpp remodeling. Osmotic stress triggers DD-carboxypeptidase and lytic transglycosylase (LTG) activation, supporting mechanical load bearing through PG–OM coupling. Temperature stress (cold shock) activates LDTs, increasing 3–3 CLs. pH stress activates a distinct set of DD-carboxypeptidases and LTGs, preserving cell morphology and maintaining PG homeostasis. Antibiotic stress (β-lactams) triggers coordinated activation of DD-carboxypeptidase, LDTs, LTGs, and glycosyltransferase, enabling a 4–3 CLs bypass mechanism that confers antibiotic resistance or tolerance. Nutrient limitation activates the stringent response, decreasing PG synthesis. Finally, oxidative and chemical stress inhibit LDT activity, decreasing overall CLs. PG: peptidoglycan; OM: outer membrane; LPS: lipopolysaccharide; LDT: LD-transpeptidase; LTG: lytic transglycosylase; CLs: cross-links; Lpp: Braun’s lipoprotein. Created in BioRender. Strohhammer, T. (2026), https://BioRender.com/regmp4c (accessed on 31 July 2026).

The importance of PG remodeling becomes evident when bacterial growth slows or ceases (Figure 2). As cells transit from active proliferation to a non-proliferation state, cell wall metabolism shifts from supporting envelope expansion to preserving structural integrity. This transition enables bacteria to withstand prolonged stress, maintain envelope homeostasis, and retain the capacity to resume growth once favorable conditions are restored. Accordingly, the transition to stationary phase represents a major shift in cellular priorities, where growth-associated PG expansion is reduced and maintenance, restructuring, and stress adaptation become dominant. Under these conditions, the dynamic interplay between PG synthases and hydrolases is reprogrammed, leading to measurable alterations in PG composition, cross-linking, and turnover.

A central regulator coordinating this physiological transition is the alternative sigma factor σ^S^ (RpoS). In *E. coli*, RpoS functions as the master regulator of the general stress response and is induced as cells enter stationary phase in response to nutrient limitation, reduced growth rate, and the accumulation of stress signals such as reactive oxygen species [45,46,47]. The RpoS regulon encompasses a large set of genes involved in stress resistance, metabolic adaptation, and envelope maintenance, accounting for an estimated 10% of the genome [48]. RpoS-dependent pathways have been shown to alter PG architecture during stationary phase, leading to increased cross-linking, enhanced coupling between the PG and OM, and reduced envelope permeability, thereby strengthening the cell envelope under stress conditions [49,50]. Within this regulatory framework in *E. coli*, RpoS directly and indirectly controls several envelope-associated and PG remodeling factors, including PBPs, cell division proteins like FtsAQZ and LD-transpeptidases [51,52,53]. LdtE, the LDT which catalyzes the formation of 3–3 cross-links, is part of the RpoS regulon and primarily expressed in stationary phase [53]. This leads to an increase in 3–3 cross-links compared to exponentially growing cells, indicating a modulation of overall cross-links within the cell wall. While in exponentially growing cells 3–3 cross-links represent ~3% of total cross-links, the level of 3–3 cross-links rises to ~10% in stationary phase [54,55]. Additionally, DpaA, is also under RpoS control and is maximally expressed in stationary phase, where it has been demonstrated to stimulate 3–3 cross-link production through LdtE [53,56]. A similar growth-phase dependence of 3–3 cross-link formation has been observed in *Pseudomonas aeruginosa*, where 3–3 cross-links are absent during exponential growth but accumulate in stationary phase and during biofilm formation, suggesting that LDT-driven PG remodeling during growth arrest is conserved across several Gram-negative lineages examined to date [57]. However, the physiological role of 3–3 cross-links increase in stationary phase cells is not known yet.

Stationary phase is further characterized by regulatory mechanisms that directly modify PG composition beyond cross-linking, in order to adapt the cell envelope to non-growth conditions. A key example is the production of non-canonical D-amino acids (NCDAAs), which accumulate during stationary phase and are incorporated into the PG polymer by LD-transpeptidases, replacing the terminal D-alanine of stem peptides [58,59]. Beyond their direct structural role, NCDAAs participate in a regulatory feedback circuit linking PG recycling to synthesis. During PG turnover in *Vibrio cholerae*, NCDAA-modified anhydro-muropeptides are released from the sacculus and re-imported into the cytoplasm via AmpG permease. There, they accumulate as tetrapeptide precursors because LD-carboxypeptidases preferentially process canonical D-alanine-terminated substrates over NCDAA-modified ones. This substrate selectivity is proposed to cause NCDAA-modified tetrapeptides to accumulate in stationary phase, which in turn downregulates PG synthesis at two levels: by reducing DD-cross-links through incorporation as acceptor-only substrates, and by competing with the de novo synthesis pathway for shared UDP-MurNAc precursors [60]. The *dadX*-encoded alanine racemase contributes to this regulatory circuit by providing D-alanine substrate availability, which feeds both canonical PG precursor synthesis and NCDAA production, thereby coupling cell wall remodeling to the metabolic state of the cell during growth arrest [61].

The principle that growth arrest drives PG remodeling extends beyond stationary phase per se. In non-proliferating intracellular *Salmonella enterica* serovar Typhimurium, a physiological state sharing key features with stationary phase including low growth rate, stress signaling, and LdtE upregulation, PG undergoes substantial remodeling, where 3–3 cross-links increase by ~30%, glycan chains shorten, and an unprecedented modification arises in which the terminal D-alanine of stem peptides is replaced by the amino alcohol alaninol. This chemical edit attenuates NF-κB-mediated immune signaling, suggesting that PG remodeling in non-proliferating bacteria is consistent with an immune-evasion function [62]. A similar remodeling program is also observed during viable but non-culturable (VBNC) transition. In *E. coli,* VBNC cells undergo extensive remodeling of the PG, supporting the concept that envelope maintenance is essential for long-term survival under stress. VBNC cells become coccoid and display a markedly remodeled sacculus characterized by increased overall cross-linking, a threefold enrichment in 3–3 cross-links, increased covalent attachment of Lpp, and significantly shorter glycan strands. These changes occur despite the absence of detectable synthesis-associated high-molecular-weight PBPs, indicating that dormant cells preserve envelope integrity through selective remodeling rather than active growth-associated PG synthesis [63].

Together, these examples indicate that PG remodeling during growth arrest is not merely a passive consequence of reduced biosynthetic activity but an actively regulated process with functional consequences for envelope integrity, stress resistance, and host interactions. The resulting sacculus differs structurally from that of growing cells, reflecting an adaptation towards increased stability and maintenance rather than expansion.

## 5. Peptidoglycan Remodeling Under Stress Conditions

PG remodeling is a broader adaptive response (Figure 2). The remodeling pathways activated under environmental stress largely overlap with those engaged during stationary phase, reinforcing the concept that PG remodeling may be considered a general survival strategy.

### 5.1. Environmental Adaptation

The primary function of PG is to resist the mechanical forces generated within the bacterial cell while preserving envelope integrity. This requires a delicate balance between rigidity, which prevents osmotic rupture, and plasticity, to allow cell growth and morphogenesis.

#### 5.1.1. Osmotic Shock

Turgor pressure, the outward force exerted by the cytoplasm on the cell wall, imposes a constant mechanical load on the PG sacculus and must always be balanced to prevent lysis. Hyperosmotic shock reduces turgor and causes plasmolysis, while hypoosmotic shock increases it and may result in lysis. The rate of PG synthesis is coupled to sacculus pore dimensions, turgor and tension. This control is exerted by the OM anchored lipoproteins LpoA and LpoB which are activators of the major bifunctional PG synthases PBP1A and PBP1B, respectively, and were independently shown to form specific trans-envelope complexes with their cognate PBPs, stimulating their transpeptidase activity from the outer face of the sacculus [27,64]. LpoA was demonstrated to not only activate the transpeptidase function of PBP1A, but also to stimulates PG polymerization through PBP1A’s glycosyltransferase activity [65]. In line with its regulatory role of the major PBP involved in elongation, it localizes at the sidewall of growing cells [66]. LpoB also promotes glycan chain polymerization by PBP1B and its OM anchoring enables it to regulate PG synthesis rate in response to the mechanical state of the sacculus. By spanning the periplasm and threading through PG pores to reach PBP1B, LpoB activity is sensitive to pore size, coupling PG synthesis rate to turgor and overall cellular growth rate [67]. More recently, single-molecule studies revealed that LpoB activates PBP1B through a transient allosteric interaction, which triggers conformational rearrangements to initiate synthesis before dissociating and directing PG synthesis to areas of low PG density [68].

Under hyperosmotic conditions, reduced turgor shrinks PG pores, limiting both LpoA and LpoB’s ability to thread through the sacculus and activate their cognates PBPs, thereby reducing PG synthesis rate in response to the osmotic shift [67]. Under hypoosmotic conditions, the PG-OM unit itself functions as a pressure vessel where the attachment of Lpp to PG limits periplasmic volume expansion, OmpA acts as a rigidifying clamp, and the Tol-Pal complex functions as an active tension-maintaining motor. Together, these passive tethers and active motors secure the OM to the PG mesh, generating periplasmic counter-pressure that prevents lysis during osmotic expansion [69]. Indeed, biophysical measurements have shown that the OM and PG function as a mechanical unit rather than independent layers. OM-PG attachment limits periplasmic volume expansion under hypoosmotic shock, generating periplasmic pressure that balances cytoplasmic turgor across the IM and prevents lysis, demonstrating that PG alone is not sufficient to maintain turgor and that the integrity of the entire envelope unit is required [69,70]. The primary adaptation to osmotic stress thus operates at the mechanical rather than the enzymatic level, with the structural properties of the sacculus and its coupling to the OM providing the first line of adaptation.

#### 5.1.2. Adaptation to pH Changes

Conversely, pH stress engages a dedicated enzymatic remodeling response through specialized DD-carboxypeptidases, LTGs and LDTs. PBP6b’s stability and its substrate affinity are enhanced at low pH, and its deletion causes increased pentapeptide content in PG and morphological defects under acidic growth conditions [35]. The LTGs MltE and MltC are similarly induced at acidic pH, MltE showing increased enzymatic activity and MltC showing increased protein levels. Their combined deletion causes growth defects, chaining morphology indicative of failed daughter cell separation, and increased antibiotic susceptibility specifically under acidic conditions [71]. At low pH also the activity of LdtE is enhanced [56] suggesting that the relative abundances of different PG cross-linking types might be regulated to adapt to several environmental conditions. At alkaline pH, the DD-carboxypeptidase PBP6a is stabilized and activated, interacting physically with other PBPs (PBP1a, PBP1b, PBP2, and PBP3) which are required for cell shape maintenance. PBP6a and PBP5 are required for growth under alkaline stress, while PBP5 alone is required under salt stress [72]. Together these findings reveal that a subset of specialized enzymes allows the cell to maintain PG homeostasis across a broad range of chemical environments. Temperature stress adds a further dimension: during cold shock the cold-inducible RNA helicase DeaD, facilitates the translation of *ldtD* mRNA leading to the increase of 3–3 cross-links, thus contributing to bacterial survival and fitness during cold stress [73].

#### 5.1.3. Nutrient Stress

Cell wall modification is also induced under nutrient limitation through the stringent response, a global bacterial adaptation mediated by the alarmone (p)ppGpp that reallocates cellular resources from growth to survival during nutrient starvation and environmental stress [74,75]. Physiological concentrations of (p)ppGpp alarmone inhibit lipid II synthesis and PG polymerization in vitro [76]. When OM biogenesis is perturbed, (p)ppGpp production causes cells to enter a semi-dormant state, whereas (p)ppGpp-lacking cells exposed to the same perturbation continue to grow uncontrollably and lyse, demonstrating that the stringent response coordinates growth in response to OM biogenesis defects and is required to prevent lysis [77]. Beyond its effect on PG synthesis rate, (p)ppGpp controls cell size and division timing, with ppGpp level rather than growth rate determining the steady-state cell size and the amount of growth added per cell cycle [78]. The stringent response thus functions as a global coordinator that recalibrates PG metabolism, reducing synthesis and adjusting division timing until biosynthetic capacity is restored. An example for the effect of nutrient limitation and its effect on PG remodeling was demonstrated by Alvarez and colleagues [79]: Cell cultures of *V. cholerae* grown in M9 minimal media with different carbon sources show higher 3-3 cross-linking and subsequent lower anhydro-muropeptide levels, indicating that 3–3 cross-links restrict accessibility of tranyglycosylases to the substrate.

#### 5.1.4. Oxidative and Chemical Stress

Stresses that act through direct chemical interference with PG enzymes or PG, including reactive oxygen species and toxic compounds, represent a distinct category of challenge to PG integrity, one that operates not by altering the mechanical load on the sacculus but by disrupting the enzymatic machinery that maintains it. Hydroxyl radical overproduction specifically within the envelope compartment of *E. coli* directly or indirectly arrests PG synthesis and causes cell lysis, a finding that implicates oxidative stress in the envelope as an important and previously underappreciated stressor of PG metabolism, distinct from the well-established effects of cytoplasmic reactive oxygen species on DNA [80]. In the gastric pathogen *Helicobacter pylori*, oxidative stress upregulates the PG deacetylase HP310 which removes acetyl group from GlcNAc in the glycan backbone and confers resistance to lysozyme, demonstrating that bacteria can remodel PG composition in response to chemical stress likely as a mechanism to escape immune detection [81]. Copper provides a further example of chemical interference with PG enzyme activity: sub-inhibitory concentrations of copper chloride reduce 3–3 cross-linking by inhibiting LD-transpeptidases in *E. coli*, making cells unable to survive to LPS transport blockage and demonstrating that chemical inhibition of specific PG enzymes can compromise the cell’s capacity to respond to other concurrent envelope stresses [82].

Across the stresses that cells can face, a common logic emerges: the cell maintains a repertoire of enzymatically specialized PG-modifying proteins, synthases, cross-linking enzymes, carboxypeptidases and hydrolases, that are selectively activated or stabilized as conditions change, allowing finetuned PG biosynthesis and remodeling (Figure 2). Rather than maintaining a single fixed enzymatic configuration, bacteria exploit the redundancy of their PG enzyme repertoire to continuously adjust sacculus composition and synthesis rate to prevailing conditions, a capacity that underpins bacterial resilience across the full range of the environments they inhabit.

### 5.2. Loss of OM Asymmetry

Severe OM stress also induces a reversible reduction in growth that resembles other non-growing physiological states. Under these conditions, bacteria rely on dedicated PG remodeling programs to stabilize the envelope until membrane biogenesis can resume (Figure 2). As outlined above, the stability of the Gram-negative cell envelope depends not only on the mechanical properties of the PG sacculus but on its covalent linkage with the OM, a connection maintained in large parts by Lpp, providing the only covalent connection between these two layers [11]. This tether sets the width of the periplasm and contributes directly to the mechanical stiffness of the OM [10]. The OM itself has also been recognized as a load bearing structure which together with the PG counteracts the turgor pressure [70]. Several examples highlight this functional interaction.

**Figure 3:**
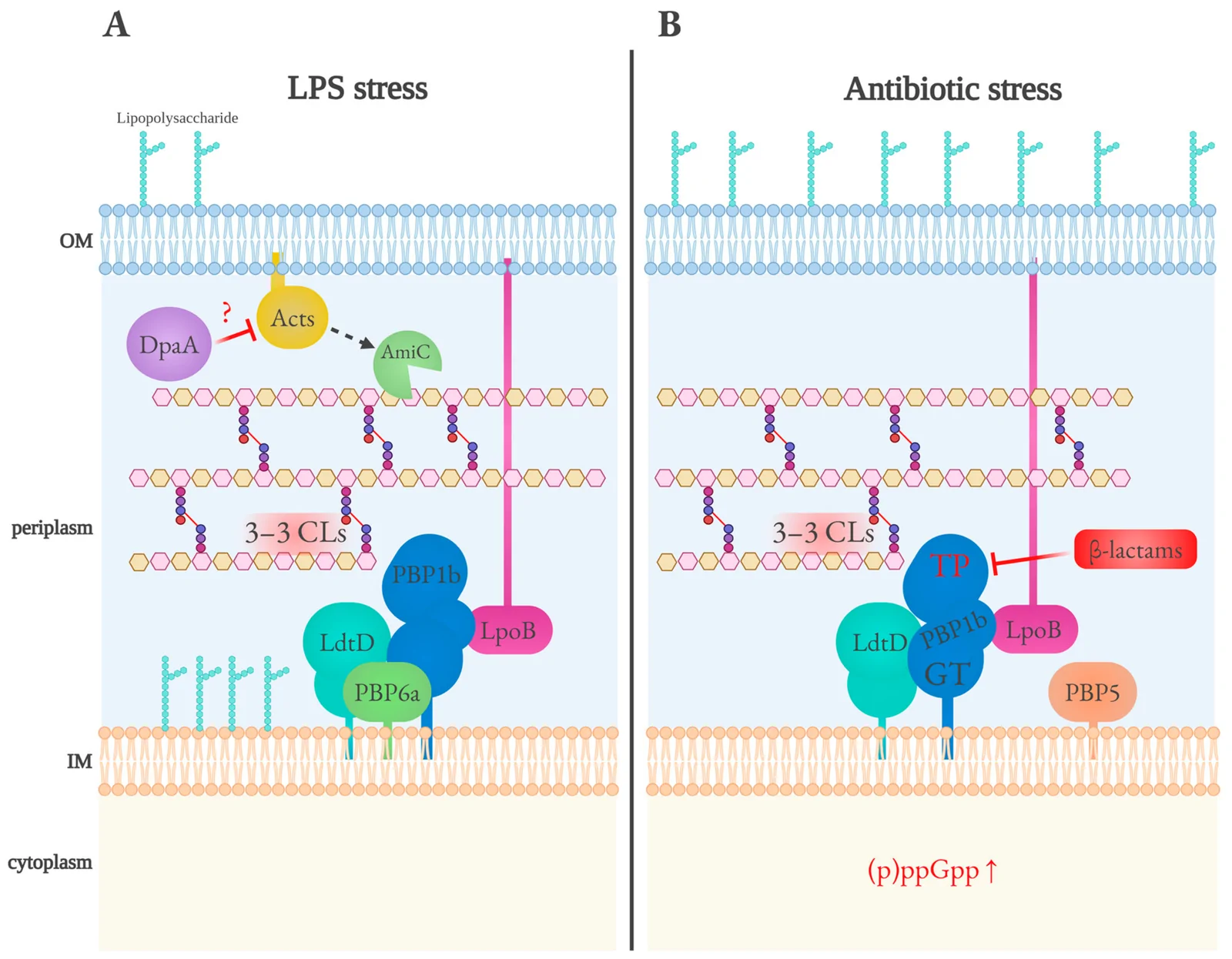
Distinct PG remodeling responses to LPS transport defects and β-lactam antibiotic stress. Schematic of the Gram-negative cell envelope (outer membrane, OM; periplasm; inner membrane, IM; cytoplasm) depicting peptidoglycan (PG) stress-response programs. (**A**) Lipopolysaccharide (LPS) transport defects. When LPS export to the cell surface is blocked (e.g., upon defects of the Lpt transport machinery), LPS accumulates at the IM outer leaflet instead of reaching the OM. To preserve envelope integrity, a membrane-anchored repair complex assembles locally: PBP6a (DD-carboxypeptidase) trims stem peptides to the tetrapeptide form, PBP1b polymerizes new glycan strands together with its OM-anchored activator LpoB, and the LD-transpeptidase LdtD introduces 3–3 cross-links (3–3 CLs) into the newly made PG. In parallel, DpaA restrains the amidase activator ActS, which activates amidases, preferentially AmiC, through a mechanism that remains unknown (red inhibitory arrow, “?”). In the absence of DpaA, ActS spuriously activates AmiC, which removes the stem peptides from glycan strands, leading to uncontrolled PG hydrolysis and cell lysis. (**B**) β-lactam antibiotic stress. β-lactams inhibit the DD-transpeptidase (TP) domain of PBP1b (indicated in red) preventing conventional 4–3 CLs formation. To bypass, PBP5 supplies the tetrapeptide substrate required by LdtD, which forms 3–3 CLs. Glycan strand polymerization is carried out by the glycosyltransferase (GT) domain of PBP1b, which remains active and continues to associate with LpoB even though PBP1b’s TP domain is enzymatically inactivated under β-lactam exposure, indicating that LpoB directly stimulates PBP1b’s GT activity independently of its canonical role in activating transpeptidation. Elevated level of (p)ppGpp is required for full β-lactam resistance. Created in BioRender. Strohhammer, T. (2026). https://BioRender.com/mgfg1bj (accessed on 31 July 2026).

When LPS transport to the OM is severely compromised, *E. coli* activates a dedicated PG remodeling program that is essential for survival. LPS transport to the cell surface is ensured by the Lpt protein machinery, comprising seven essential core components (LptABCDFG) which assemble to form a transenvelope complex [83]. Upon conditional depletion of LptC [84], cells block LPS transport, go into growth arrest and form short chains but remain viable for several hours and resume growth upon restoration of LptC expression. This survival mechanism requires a rapid and specific increase in 3–3 cross-links in the PG sacculus [53]. Proteomics has previously shown an increased abundance of PBP1B, PBP5, and PBP6a among other envelope-related proteins in LptC depleted cells [85], and Morè and colleagues [53] demonstrated that this upregulation is functionally essential: cells lacking LDTs responsible for 3–3 cross-links lyse upon LptC depletion, whereas cells lacking only the Lpp-attachment LDTs (LdtA, LdtB, LdtC) do not, identifying 3–3 cross-link formation specifically as the protective mechanism. In this remodeling process the glycosyltransferase activity of PBP1B and its OM-anchored activator LpoB, as well as the DD-carboxypeptidase PBP6a work in complex with LdtD ensuring cell survival, whereas the activity of PBP1A, LpoA, and PBP5 is not required (Figure 3A) [53]. This specificity is noteworthy: it distinguishes the OM stress response from the β-lactam bypass mechanism identified by Hugonnet and colleagues [55], in which LdtD (YcbB) in vivo cooperates with PBP1B and the DD-carboxypeptidase PBP5 rather than PBP6a plus (p)ppGpp/RelA to achieve broad-spectrum β-lactam resistance, underscoring that these are related but distinct enzymatic configurations of the same core machinery deployed under different stress conditions (Figure 3B). In the OM stress context, PBP1B/LpoB polymerizes new glycan strands via its glycosyltransferase activity, PBP6a trims the resulting pentapeptides to tetrapeptides, and LdtD cross-links the tetrapeptides via 3–3 cross-links, preferentially acting on nascent rather than pre-existing PG [53]. A further dimension of this remodeling program is provided by DpaA, the enzyme that detaches Lpp from the sacculus; its essentiality in cells with impaired LPS export reveals that the OM-PG connection is subject to active remodeling under stress [22]. DpaA also indirectly controls the activity of ActS, an OM-anchored lipoprotein with a degenerate catalytic LytM domain that does not exhibit endopeptidase activity but in vitro activates all three periplasmic amidases, AmiA, AmiB and AmiC [86,87]. However, in the cell, ActS activity is required under OM stress conditions. In the absence of DpaA, ActS spuriously activates AmiC, causing morphological defects and lysis when LPS transport is compromised (Figure 3A). Deletion of *actS* suppresses this lysis, although the mechanism by which DpaA restrains ActS activity remains unknown but is proposed to involve altered PG architecture rather than direct protein–protein interaction [86]. The physiological importance of LDT activity under OM stress is further underscored by the finding, outlined in the previous chapter, that sub-inhibitory concentrations of copper chloride inhibit LDTs, and make cells unable to survive LPS transport blockage [82].

Two models have been proposed to explain why 3–3 cross-links rescue cells from lysis, when OM is compromised and both are consistent with the available data. The first is mechanical: defects that may perturb OM asymmetry and reduce its load-bearing capacity, creating local mechanical stress on the underlying PG that LDT-mediated remodeling could compensate by strengthening the sacculus. The second is structural where the Lpt transenvelope bridge disassembles upon LptC depletion, leaving gaps in the PG where the machinery was threaded, and the PBP1B–LdtD–PBP6a triad closing these gaps by synthesizing PG enriched in 3–3 cross-links [53,70].

The broader conservation of this PG–OM coupling mechanism is illustrated by studies in *Acinetobacter baumannii*. In this organism LPS is not essential [88] but its loss, which occurs following selection of mutants resistant to colistin, requires compensatory mutations that lower the expression of PBP1A [89]. Additionally, LdtJ, a YkuD family member responsible for 3–3 cross-links incorporation in the PG is also required for survival in cells devoid of LPS, following colistin selection, and becomes essential in cells lacking PBP1A [90]. Notably, deletion of *ldtJ*, disrupts cell morphology, downregulates PG precursors genes and activate the stringent response. These defects are suppressed in a mutant in which the Mla pathway is inactivated [91]. The Mla system is an ABC transporter that maintains OM asymmetry by transporting misplaced phospholipids from the OM outer leaflet back to the IM [92], thus revealing a functional interplay between PG remodeling and OM lipid homeostasis. These concepts are further expanded by studies showing lipid asymmetry as a structural checkpoint controlling access to lipooligosaccharide (LOS)-independent survival. Disruption of phospholipid transport and surface phospholipid degradation destabilizes membrane balance, creating a permissive state that enables the emergence of LOS-deficient, colistin-resistant variants which are stabilized by repression of PBP1A [93]. Therefore, in *A. baumannii* a complex molecular network coordinates phospholipid homeostasis, PG remodeling, and stabilization of LOS-deficient envelope state.

## 6. Peptidoglycan Remodeling Under Antibiotic Stress, Tolerance and Resistance

The PG remodeling programs engaged during growth arrest, non-growing physiological states, and OM defects are not confined to these conditions: several of the same structural outputs, like increased cross-linking, altered hydrolase activity, and envelope-OM reinforcement, recur when cells encounter cell-wall-targeting antibiotics, suggesting a shared molecular logic linking envelope adaptation under growth arrest to survival under antibiotic pressure.

### 6.1. Antibiotic Tolerance

Antibiotics targeting PG synthesis constitute an important class of clinically useful molecules which challenge the fundamental balance between synthesis and hydrolysis that maintains cell envelope integrity and ultimately cell survival [94,95]. The focus of this chapter is on β-lactams as the most important and largest class, while glycopeptides are not considered as they are generally only active against Gram-positive bacteria [96]. The mode of action of β-lactams in Gram-negative bacteria has been largely studied in *E. coli*, where rapid lysis is observed when a class A and at least one class B PBP are simultaneously inhibited, leading to PG synthesis inhibition and hydrolase activation [97]. PG degradation products from lytic transglycosylases and endopeptidases accumulate in β-lactam-treated *E. coli* cells, and cell division amidases appear to be the major contributors to lysis [94,98,99]. The mechanism of β-lactam lethality was more recently revisited by Cho and colleagues [100], who showed that transpeptidase inhibition by mecillinam generates long uncross-linked PG strands which are degraded by the lytic transglycosylase Slt in a futile cycle of PG synthesis and degradation, depleting cellular resources and ultimately contributing to cell death.

Bacteria can survive β-lactam treatment if they possess or can express resistance determinants. Alternatively, they can remain viable but unable to grow for extended periods after exposure to the antibiotic and then remain able to resume growth once the antibiotic is removed, a trajectory defined as antibiotic tolerance [101]. Several clinically significant Gram-negative pathogens are remarkably tolerant to β-lactams. *V. cholerae* survives exposure to cell wall synthesis inhibitors by forming non-dividing, cell wall-deficient spheroplasts that readily revert to normal rod morphology upon antibiotic removal [102]. This tolerance depends on the VxrAB two-component system, previously named WigKR, which is activated by cell wall damage and controls a broad regulon encompassing the entire cell wall synthesis pathway [103]. How VxrAB promotes high-level tolerance is not completely understood but presumably relies on the repair of cell envelope damage inflicted by β-lactam antibiotics. More recently VxrAB activation has also been shown to downregulate iron uptake transporters, reducing antibiotic-induced toxic iron accumulation and enhancing tolerance in a mouse model of cholera infection [104]. VxrAB thus defines a signal transduction pathway controlling PG biosynthesis and antibiotic tolerance through distinct but synergistic mechanisms [103,104].

PG remodeling through proteolytic control defines another mechanism that contribute to β-lactam tolerance in *E. coli*. Loss of Prc, a periplasmic protease that regulates several hydrolases at post-translational level, not only compromises envelope integrity but also reduces the formation of *E. coli* persistent cells in response to diverse cell-wall-targeting antibiotics [105].

Genome-wide approaches have extended these findings across organisms. Transposon insertion sequencing in *V. cholerae* identified cell wall and LPS biogenesis genes and the σ^E^ envelope stress response as critical determinants of recovery from β-lactam treatment [106]. In *E. coli*, transposon-directed insertion sequencing coupled to inducible gene expression highlighted PG remodeling during cell division, cell envelope synthesis and maintenance, and ATP metabolism as relevant for meropenem tolerance [107]. In *Burkholderia* spp., the top determinants of meropenem tolerance included immunity to a type VI toxin targeting PG, PG recycling, and the σ^E^ stress response [108]. Together, these observations reinforce a view of tolerance as active physiological adaptation rather than passive dormancy.

### 6.2. Antibiotic Resistance

The most striking example of PG remodeling linked to β-lactam resistance is the LD-transpeptidase bypass pathway in *E. coli*. When classical PBPs are inhibited by β-lactams, LdtD, together with PBP1B, PBP5, can bypass DD-transpeptidation and produce PG in which stems peptides are exclusively linked by 3–3 cross-links. Notably, increased synthesis of the alarmone (p)ppGpp by RelA is required for increased LdtD activity and broad-spectrum β-lactam resistance [55]. A related but distinct example is provided by PBP5, encoded by *dacA*: by removing the terminal D-Ala residue from uncross-linked stem peptides, PBP5 modulates the overall crosslinking level in the PG [3], and *dacA* deletion increases sensitivity to several β-lactam antibiotics. The physical interaction of PBP5 with DD-transpeptidases, rather than its catalytic activity, is responsible for this effect on β-lactam susceptibility. Notably, in *dacA* deleted cells resistance to the glycopeptide vancomycin is increased due to elevated amount of its target, the D-Ala-D-Ala residues in the PG, which act as a decoy for the antibiotic [109]. A similar seesaw effect can be observed when deleting the *slt* and *mltG* LTG in *E coli*: their loss leads to increased susceptibility to β-lactam antibiotics through a mechanism dependent on LTG enzymatic activity, while simultaneously conferring vancomycin resistance through a PBP5-dependent mechanism whose precise molecular basis remains to be directly demonstrated. These data suggest that LTG-targeting strategies, while attractive for β-lactam potentiation, must be evaluated in the context of this reciprocal resistance liability, though in practice, vancomycin is not used for Gram-negative infections owing to its inability to penetrate the OM [110].

## 7. Targeting Peptidoglycan Remodeling Pathways for Antibiotics and Adjuvant Development

PG synthesis has always been a prominent target for antibiotics due to conservation among Gram-negative bacteria and absence of this pathway in the human counterpart. Instead, targeting PG remodeling pathways has gained substantial momentum in recent years, and the growing appreciation of the enzymatic complexity of PG metabolism has revealed multiple potential targets, including PG synthesis enzymes, hydrolases, and their regulators [111,112].

### 7.1. Targeting Lytic Transglycosylases

LTGs are the best-developed case for periplasmic PG glycosylase targeting. In *E. coli*, eight LTGs have been identified, and individual deletions are well tolerated due to their high functional redundancy, a property that long obscured their consideration as antibiotic targets [113]. However, multiple LTG deletions cause pronounced growth defects, daughter cell separation failure, and altered antibiotic susceptibility profiles, whereas individual LTGs are dispensable, showing that their collective activity is required for proper envelope homeostasis [71,110]. The mechanistic basis for this collective essentiality was established in *V. cholerae*, where a conditional LTG-null mutant revealed that the primary essential function of LTGs is not space-making for new PG insertion, as long assumed, but the clearance of soluble, endopeptidase-generated uncross-linked PG strands from the periplasm. This constitutes a function whose disruption causes periplasmic crowding and acute sensitivity to hypoosmotic conditions [114].

The structural conservation of the LTG active site across diverse Gram-negative bacteria makes this class of enzymes appealing for broad-spectrum inhibitor design. All LTGs share a conserved catalytic glutamate or aspartate that performs acid-base catalysis to cleave the β-1,4-glycosidic bond between MurNAc and GlcNAc with concomitant formation of a 1,6-anhydro bond in the MurNAc residue. This active site architecture is sufficiently conserved so that a single inhibitor, bulgecin A, a natural product of *Pseudomonas mesoacidophila*, can inhibit structurally diverse LTGs from *E. coli*, *Neisseria meningitidis*, and *H. pylori* by binding the conserved substrate-binding groove [113,115,116]. Extracts containing bulgecin A can restore susceptibility to carbapenem of *P. aeruginosa* and *A. baumannii* carbapenem resistant strains [117]. Bulgecin A inhibition of LTGs was proposed to enhance β-lactam activity by preventing clearance of the uncross-linked glycan strands that β-lactams generate upon transpeptidase inhibition, causing them to accumulate and drive structural failure of the cell wall [100,116,118]. The mechanistic basis for bulgecin–β-lactam synergy became clearer with the discovery that β-lactams do not simply inhibit PBP transpeptidase activity but actively derange the PG biogenesis machinery [116,118]. Bulgecin A combined with sub-minimum inhibitory concentrations (MIC) of ceftazidime results in an early onset of bactericidal activity against *P. aeruginosa*, *Enterobacter aerogenes*, and *A. baumannii*, with the accelerated killing kinetics critical for rapid reduction in bacterial load in an infection [118]. The clinical relevance of bulgecin A is also demonstrated by its ability to restore the efficacy of penicillin G and amoxicillin in strains of *Neisseria meningitidis* and *N. gonorrhoeae* with reduced β-lactam susceptibility, lowering MICs below clinical susceptibility breakpoints [116]. However, targeting LTGs poses significant limitations. Despite structural conservation, LTG redundancy means that inhibiting a single enzyme is unlikely to be sufficient [114]. Therefore, simultaneous impairment of functionally complementary enzymes is required for efficacy, complicating inhibitor strategies. Also, it has been shown that simultaneous LTG deletion sensitizes to β-lactams but it may confer vancomycin resistance through a PBP5-dependent mechanism, introducing a limitation that requires organism-specific evaluation [109,110]. In the context of Bulgecin A it has been shown that this natural product accumulates in the periplasm of *P. aeruginosa* at levels 67-fold lower than ciprofloxacin, and 40-fold lower in *E. coli*, with OM penetration barrier accounting for the high concentrations required for potentiation activity, a constraint that likely explains the absence of potentiation observed against *Klebsiella* spp. [111,118,119]. This explains why no LTG inhibitor has entered clinical development yet.

### 7.2. Targeting Peptidases

Endopeptidases represent a mechanistically distinct target class with a different rationale. This class of proteins is needed for cell elongation, and functional redundancy has been detected among the enzymes that catalyze cleavage of peptide bonds in PG [32].

Structure-based inhibitors have been successfully developed against Pgp3 from *Campylobacter jejuni*, a M23/LytM-family protein with both DD-endopeptidase and DD-carboxypeptidase function. Hydroxamate-based compounds that chelate the active-site Zn^2+^ ion competitively inhibited Pgp3, reverted the bacterium’s helical morphology, and reduced host–cell invasion, demonstrating that rationally designed inhibitors targeting this enzyme family can produce functional consequences for cell shape and virulence [43]. While Pgp3 combines endopeptidase and carboxypeptidase activities, other PG peptidases shape bacterial morphology through carboxypeptidase activity alone. In *H. pylori*, the cell-shape determinant Csd4 is a DD-carboxypeptidase; loss of *csd4* impairs *H. pylori* motility, helical cell shape, and colonization of the host stomach [44]. Structure-guided design based on the Csd4 active site yielded an inhibitor that recapitulated the *csd4*-deletion phenotype, altering cell shape and viability, although only at high millimolar concentrations [120]. These examples support PG peptidases as a mechanistically validated but still an early-stage class of antibacterial targets, with potency and in vivo translatability remaining the key hurdles to clinical development.

## 8. Conclusions and Outlook

PG remodeling is the central homeostatic hub of the Gram-negative cell envelope. The sacculus is continuously remodeled by a repertoire of synthases, cross-linking enzymes, carboxypeptidases, endopeptidases and hydrolases whose activities are spatially and temporally coordinated across the cell cycle and calibrated in response to the mechanical, chemical, and nutritional state of the cell. This coordination is not incidental, it is the mechanism by which bacteria maintain structural integrity while simultaneously growing, dividing, and adapting to environmental changes. The capacity to maintain synthesis-hydrolysis balance under stress determines survival, and the conservation of these processes across phylogenetically diverse Gram-negative bacteria argues that PG remodeling is a fundamental property of the envelope. Growth arrest, OM perturbation, osmotic shifts, pH, temperature, nutrient limitation, oxidative challenge, and antibiotic exposure each engage distinct but overlapping configurations of the same core enzymatic machinery, revealing a division of labor that allows the cell to tailor its response to each specific condition. The same hydrolases essential for growth become instruments of lethality when synthesis is blocked, a paradox that defines both the challenge of antibiotic resistance and the opportunity for therapeutic exploitation. The role of PG remodeling in tolerance to cell-wall-targeting antibiotics and in survival of VBNC cells highlights this process not as a passive consequence of growth arrest but as a mechanism to preserve envelope functionality in otherwise lethal conditions. Altogether, these observations reinforce the concept that extensive PG remodeling is important during growth, but central under non-growing physiological states and likely contributes to the cell’s remarkable capacity to survive prolonged environmental and antibiotic stress.

Antimicrobial resistance represents one of the most pressing global health challenges of our time, and the pipeline of novel antibiotics has failed to keep pace with the emergence of resistant pathogens [121]. All cell wall-active antibiotics currently used for Gram-negative pathogens like *V. cholerae*, *H. pylori* or *N. gonorrhoeae* target PG synthesis rather than remodeling, leaving the autolytic side of the synthesis-remodeling balance entirely unexploited therapeutically. The findings reviewed here suggest that this gap represents not merely an oversight but an opportunity: PG remodeling enzymes are mechanistically validated targets whose inhibition, particularly in combination with existing β-lactams, could restore efficacy against resistant organisms.

PG remodeling enzymes offer opportunities for developing antibiotic adjuvants. LTG or peptidase inhibitors combined with β-lactams, exploit the futile cycle mechanism and periplasmic crowding principle without requiring standalone activity against resistant organisms, potentiating antibiotics already in widespread clinical use while targeting mechanistic weaknesses that resistance mutations cannot easily circumvent. Yet direct pharmacological targeting of peptidases remains substantially underdeveloped relative to LTG inhibition: the Pgp3 and Csd4 inhibitors described above remain among the only examples with demonstrated pharmacological activity in a cell-based or whole-organism context. The challenge is translating these mechanisms into molecules with adequate pharmacological properties, particularly OM penetration, and multi-target strategies that overcome enzymatic redundancy. However, in the last few years generative artificial intelligence models can overcome these challenges by exploring new molecular sequences that occupy regions of antimicrobial space not yet represented in nature or existing libraries. This approach can embed multi-objective constraints, including potency, selectivity, penetration across membrane, low toxicity, and resistance risk, directly into the design process rather than applying them only as downstream filters [122]. Also, the current understanding of how PG remodeling factors are coordinated in space and time within the periplasm is incomplete and dissecting this aspect is not only of fundamental interest but a prerequisite for rational exploitation of regulatory nodes that this review identifies as therapeutically relevant. 

The most compelling proof of concept that PG hydrolases are pharmacologically accessible comes from Gram-positive biology: glycopeptide antibiotics that inhibit the full spectrum of PG hydrolases by binding PG directly and physically restricting hydrolase access to the sacculus, achieve in vivo efficacy against drug-resistant Gram-positive pathogens with low resistance development [123]. Whether analogous PG-binding strategies can be extended to Gram-negative hydrolases, whose homologues are structurally conserved but operate in a fundamentally different envelope context, remains an open and compelling question for future drug discovery.

## Figures and Tables

**Table 1:** PG-cleaving enzymes discussed in this review.

Class	Enzyme (Organism)	Function/Substrate
Glucosaminidases		Cleave the β-1,4-glycosidic bond between GlcNAc and MurNAc (hydrolytic) [29]
Muramidases		Cleave the β-1,4-glycosidic bond between GlcNAc and MurNAc (hydrolytic) [29]
Lytic transglycosylases (LTGs)	Slt, Mlts (*E. coli*)	Cleave the MurNAc–GlcNAc bond non-hydrolytically, generating 1,6-anhydroMurNAc residues; govern periplasmic glycosidase PG remodeling activity [7,8,29]
LD-endopeptidases		Cleave 3–3 cross-links (peptide bonds between stem peptides), creating space for insertion of new glycan strands [30,31,32]
DD-endopeptidases	PBP4 (*E. coli*) Pgp3 (*Campylobacter jejuni*)	Cleave 4–3 cross-links, creating space for insertion of new glycan strands [30,31,32,43]
DD-carboxypeptidases	PBP4, PBP4b, PBP5, PBP6a, PBP6b (*E. coli*) Pgp3 (*C. jejuni*) Csd4 (*Helicobacter pylori*)	Remove the terminal D-Ala from pentapeptide stems, modulating cross-link number [33,34,35,43,44]
LD-carboxypeptidases		Remove the terminal D-Ala from tetrapeptide stems, modulating cross-link number [33,34,35]
Amidases	AmiA, AmiB, AmiC (*E. coli*)	Cleave the bond between the glycan backbone and the peptide stem at the septum during cell division, ensuring daughter cell separation [36,37]

## Data Availability

No new data were created or analyzed in this study. Data sharing is not applicable to this article.

## Abbreviations

The following abbreviations are used in this manuscript:

(p)ppGpp	Guanosine tetraphosphate/pentaphosphate (alarmone)
ABC	ATP-binding cassette (transporter)
CLs	Cross-links
D-Ala	D-alanine
D-Glu	D-glutamate
GlcNAc	N-acetylglucosamine
IM	Inner membrane
L-Ala	L-alanine
LDT	LD-transpeptidase
LOS	Lipooligosaccharide
LPS	Lipopolysaccharide
Lpp	Braun’s lipoprotein
LTG	Lytic translglycosylase
*m*-Dap	meso-Diaminopimelic acid
MIC	Minimum inhibitory concentration
Mla	Maintenance of lipid asymmetry (system)
MurNAc	N-acetylmuramic acid
NCDAA	Non-canonical D-amino acid
NF-κB	Nuclear factor kappa B
OM	Outer membrane
PBP	Penicillin-binding protein
PG	Peptidoglycan
RpoS/σS	General/stationary-phase stress response sigma factor
σ^E^	Envelope stress response sigma factor
SEDS	Shape, Elongation, Division and Sporulation (protein family)
SPOR	SPOrulation-related Repeat (domain)
UDP	Uridine diphosphate
VBNC	Viable but non-culturable

## References

[1] Westblade L.F., Errington J., Dörr T. (2020). Antibiotic Tolerance. PLoS Pathog..

[2] Torrens G., Cava F. (2024). Mechanisms Conferring Bacterial Cell Wall Variability and Adaptivity. Biochem. Soc. Trans..

[3] Egan A.J.F., Errington J., Vollmer W. (2020). Regulation of Peptidoglycan Synthesis and Remodelling. Nat. Rev. Microbiol..

[4] Gupta R.S. (1998). Protein Phylogenies and Signature Sequences: A Reappraisal of Evolutionary Relationships among Archaebacteria, Eubacteria, and Eukaryotes. Microbiol. Mol. Biol. Rev..

[5] Schleifer K.H., Kandler O. (1972). Peptidoglycan Types of Bacterial Cell Walls and Their Taxonomic Implications. Bacteriol. Rev..

[6] Vollmer W. (2008). Structural Variation in the Glycan Strands of Bacterial Peptidoglycan. FEMS Microbiol. Rev..

[7] Höltje J.-V. (1998). Growth of the Stress-Bearing and Shape-Maintaining Murein Sacculus of *Escherichia coli*. Microbiol. Mol. Biol. Rev..

[8] Vollmer W., Blanot D., De Pedro M.A. (2008). Peptidoglycan Structure and Architecture. FEMS Microbiol. Rev..

[9] Aliashkevich A., Cava F. (2022). LD-transpeptidases: The Great Unknown among the Peptidoglycan Cross-linkers. FEBS J..

[10] Asmar A.T., Collet J.-F. (2018). Lpp, the Braun Lipoprotein, Turns 50—Major Achievements and Remaining Issues. FEMS Microbiol. Lett..

[11] Braun V., Wolff H. (1970). The Murein-Lipoprotein Linkage in the Cell Wall of *Escherichia coli*. Eur. J. Biochem..

[12] Benn G., Borrelli C., Prakaash D., Johnson A.N.T., Fideli V.A., Starr T., Fitzmaurice D., Combs A.N., Wühr M., Rojas E.R. (2024). OmpA Controls Order in the Outer Membrane and Shares the Mechanical Load. Proc. Natl. Acad. Sci. USA.

[13] Szczepaniak J., Holmes P., Rajasekar K., Kaminska R., Samsudin F., Inns P.G., Rassam P., Khalid S., Murray S.M., Redfield C. (2020). The Lipoprotein Pal Stabilises the Bacterial Outer Membrane during Constriction by a Mobilisation-and-Capture Mechanism. Nat. Commun..

[14] Barreteau H., Kovač A., Boniface A., Sova M., Gobec S., Blanot D. (2008). Cytoplasmic Steps of Peptidoglycan Biosynthesis. FEMS Microbiol. Rev..

[15] Bouhss A., Trunkfield A.E., Bugg T.D.H., Mengin-Lecreulx D. (2008). The Biosynthesis of Peptidoglycan Lipid-Linked Intermediates. FEMS Microbiol. Rev..

[16] Mohammadi T., Van Dam V., Sijbrandi R., Vernet T., Zapun A., Bouhss A., Diepeveen-de Bruin M., Nguyen-Distèche M., De Kruijff B., Breukink E. (2011). Identification of FtsW as a Transporter of Lipid-Linked Cell Wall Precursors across the Membrane. EMBO J..

[17] Goffin C., Ghuysen J.-M. (1998). Multimodular Penicillin-Binding Proteins: An Enigmatic Family of Orthologs and Paralogs. Microbiol. Mol. Biol. Rev..

[18] Meeske A.J., Riley E.P., Robins W.P., Uehara T., Mekalanos J.J., Kahne D., Walker S., Kruse A.C., Bernhardt T.G., Rudner D.Z. (2016). SEDS Proteins Are a Widespread Family of Bacterial Cell Wall Polymerases. Nature.

[19] Magnet S., Dubost L., Marie A., Arthur M., Gutmann L. (2008). Identification of the l,d -Transpeptidases for Peptidoglycan Cross-Linking in *Escherichia coli*. J. Bacteriol..

[20] Walenkiewicz B., Alvarez L., Cava F. (2026). LD-Transpeptidation in Bacterial Cell Walls: Biochemical Principles and Functional Diversity. Microbiol. Mol. Biol. Rev..

[21] Magnet S., Bellais S., Dubost L., Fourgeaud M., Mainardi J.-L., Petit-Frère S., Marie A., Mengin-Lecreulx D., Arthur M., Gutmann L. (2007). Identification of the L,D-Transpeptidases Responsible for Attachment of the Braun Lipoprotein to *Escherichia coli* Peptidoglycan. J. Bacteriol..

[22] Winkle M., Hernández-Rocamora V.M., Pullela K., Goodall E.C.A., Martorana A.M., Gray J., Henderson I.R., Polissi A., Vollmer W. (2021). DpaA Detaches Braun’s Lipoprotein from Peptidoglycan. mBio.

[23] Van Den Ent F., Johnson C.M., Persons L., De Boer P., Löwe J. (2010). Bacterial Actin MreB Assembles in Complex with Cell Shape Protein RodZ. EMBO J..

[24] Xiao J., Goley E.D. (2016). Redefining the Roles of the FtsZ-Ring in Bacterial Cytokinesis. Curr. Opin. Microbiol..

[25] Pazos M., Peters K., Vollmer W. (2017). Robust Peptidoglycan Growth by Dynamic and Variable Multi-Protein Complexes. Curr. Opin. Microbiol..

[26] Vermassen A., Leroy S., Talon R., Provot C., Popowska M., Desvaux M. (2019). Cell Wall Hydrolases in Bacteria: Insight on the Diversity of Cell Wall Amidases, Glycosidases and Peptidases Toward Peptidoglycan. Front. Microbiol..

[27] Typas A., Banzhaf M., Gross C.A., Vollmer W. (2012). From the Regulation of Peptidoglycan Synthesis to Bacterial Growth and Morphology. Nat. Rev. Microbiol..

[28] Vollmer W., Bertsche U. (2008). Murein (Peptidoglycan) Structure, Architecture and Biosynthesis in *Escherichia coli*. Biochim. Biophys. Acta (BBA) Biomembr..

[29] Weaver A., Taguchi A., Dörr T. (2023). Masters of Misdirection: Peptidoglycan Glycosidases in Bacterial Growth. J. Bacteriol..

[30] Meberg B.M., Paulson A.L., Priyadarshini R., Young K.D. (2004). Endopeptidase Penicillin-Binding Proteins 4 and 7 Play Auxiliary Roles in Determining Uniform Morphology of *Escherichia coli*. J. Bacteriol..

[31] Miyamoto T., Katane M., Saitoh Y., Sekine M., Homma H. (2020). Involvement of Penicillin-Binding Proteins in the Metabolism of a Bacterial Peptidoglycan Containing a Non-Canonical d-Amino Acid. Amino Acids.

[32] Singh S.K., SaiSree L., Amrutha R.N., Reddy M. (2012). Three Redundant Murein Endopeptidases Catalyse an Essential Cleavage Step in Peptidoglycan Synthesis of *Escherichia coli*
K 12. Mol. Microbiol..

[33] Nelson D.E., Young K.D. (2001). Contributions of PBP 5 and DD-Carboxypeptidase Penicillin Binding Proteins to Maintenance of Cell Shape in *Escherichia coli*. J. Bacteriol..

[34] Obando M.A., Dörr T. (2023). Novel Role for Peptidoglycan Carboxypeptidases in Maintaining the Balance between Bacterial Cell Wall Synthesis and Degradation. bioRxiv.

[35] Peters K., Kannan S., Rao V.A., Biboy J., Vollmer D., Erickson S.W., Lewis R.J., Young K.D., Vollmer W. (2016). The Redundancy of Peptidoglycan Carboxypeptidases Ensures Robust Cell Shape Maintenance in *Escherichia coli*. mBio.

[36] Uehara T., Park J.T. (2007). An Anhydro-*N*-Acetylmuramyl-L-Alanine Amidase with Broad Specificity Tethered to the Outer Membrane of *Escherichia coli*. J. Bacteriol..

[37] Uehara T., Dinh T., Bernhardt T.G. (2009). LytM-Domain Factors Are Required for Daughter Cell Separation and Rapid Ampicillin-Induced Lysis in *Escherichia coli*. J. Bacteriol..

[38] Yahashiri A., Jorgenson M.A., Weiss D.S. (2015). Bacterial SPOR Domains Are Recruited to Septal Peptidoglycan by Binding to Glycan Strands That Lack Stem Peptides. Proc. Natl. Acad. Sci. USA.

[39] Banzhaf M., Van Den Berg Van Saparoea B., Terrak M., Fraipont C., Egan A., Philippe J., Zapun A., Breukink E., Nguyen-Distèche M., Den Blaauwen T. (2012). Cooperativity of Peptidoglycan Synthases Active in Bacterial Cell Elongation. Mol. Microbiol..

[40] Hara H., Yamamoto Y., Higashitani A., Suzuki H., Nishimura Y. (1991). Cloning, Mapping, and Characterization of the *Escherichia coli* Prc Gene, Which Is Involved in C-Terminal Processing of Penicillin-Binding Protein 3. J. Bacteriol..

[41] Jeon W.-J., Cho H. (2022). A Cell Wall Hydrolase MepH Is Negatively Regulated by Proteolysis Involving Prc and NlpI in *Escherichia coli*. Front. Microbiol..

[42] Singh S.K., Parveen S., SaiSree L., Reddy M. (2015). Regulated Proteolysis of a Cross-Link–Specific Peptidoglycan Hydrolase Contributes to Bacterial Morphogenesis. Proc. Natl. Acad. Sci. USA.

[43] Choi Y., Park J.S., Kim J., Min K., Mahasenan K., Kim C., Yoon H.-J., Lim S., Cheon D.H., Lee Y. (2022). Structure-Based Inhibitor Design for Reshaping Bacterial Morphology. Commun. Biol..

[44] Sycuro L.K., Wyckoff T.J., Biboy J., Born P., Pincus Z., Vollmer W., Salama N.R. (2012). Multiple Peptidoglycan Modification Networks Modulate Helicobacter Pylori’s Cell Shape, Motility, and Colonization Potential. PLoS Pathog..

[45] Gottesman S. (2019). Trouble Is Coming: Signaling Pathways That Regulate General Stress Responses in Bacteria. J. Biol. Chem..

[46] Handler S., Kirkpatrick C.L. (2024). New Layers of Regulation of the General Stress Response Sigma Factor RpoS. Front. Microbiol..

[47] Landini P., Egli T., Wolf J., Lacour S. (2014). sigmaS, a Major Player in the Response to Environmental Stresses in *Escherichia coli*: Role, Regulation and Mechanisms of Promoter Recognition. Environ. Microbiol. Rep..

[48] Weber H., Polen T., Heuveling J., Wendisch V.F., Hengge R. (2005). Genome-Wide Analysis of the General Stress Response Network in *Escherichia coli*: SigmaS-Dependent Genes, Promoters, and Sigma Factor Selectivity. J. Bacteriol..

[49] Bouillet S., Bauer T.S., Gottesman S. (2024). RpoS and the Bacterial General Stress Response. Microbiol. Mol. Biol. Rev..

[50] Mitchell A.M., Wang W., Silhavy T.J. (2017). Novel RpoS-Dependent Mechanisms Strengthen the Envelope Permeability Barrier during Stationary Phase. J. Bacteriol..

[51] Ballesteros M., Kusano S., Ishihama A., Vicente M. (1998). The *ftsQ1p* Gearbox Promoter of *Escherichia coli* Is a Major sigma S-dependent Promoter in the *ddlB*—*ftsA* Region. Mol. Microbiol..

[52] Dougherty T.J., Pucci M.J. (1994). Penicillin-Binding Proteins Are Regulated by rpoS during Transitions in Growth States of *Escherichia coli*. Antimicrob. Agents Chemother..

[53] Morè N., Martorana A.M., Biboy J., Otten C., Winkle M., Serrano C.K.G., Montón Silva A., Atkinson L., Yau H., Breukink E. (2019). Peptidoglycan Remodeling Enables *Escherichia coli* To Survive Severe Outer Membrane Assembly Defect. mBio.

[54] Glauner B., Höltje J.V., Schwarz U. (1988). The Composition of the Murein of *Escherichia coli*. J. Biol. Chem..

[55] Hugonnet J.-E., Mengin-Lecreulx D., Monton A., Den Blaauwen T., Carbonnelle E., Veckerlé C., Brun Y.V., Van Nieuwenhze M., Bouchier C., Tu K. (2016). Factors Essential for L,D-Transpeptidase-Mediated Peptidoglycan Cross-Linking and β-Lactam Resistance in *Escherichia coli*. eLife.

[56] Montón Silva A., Otten C., Biboy J., Breukink E., VanNieuwenhze M., Vollmer W., Den Blaauwen T. (2018). The Fluorescent D-Amino Acid NADA as a Tool to Study the Conditional Activity of Transpeptidases in *Escherichia coli*. Front. Microbiol..

[57] Hugonneau-Beaufet I., Barnier J.-P., Thiriet-Rupert S., Létoffé S., Mainardi J.-L., Ghigo J.-M., Beloin C., Arthur M. (2023). Characterization of Pseudomonas Aeruginosa L,D-Transpeptidases and Evaluation of Their Role in Peptidoglycan Adaptation to Biofilm Growth. Microbiol. Spectr..

[58] Cava F., De Pedro M.A., Lam H., Davis B.M., Waldor M.K. (2011). Distinct Pathways for Modification of the Bacterial Cell Wall by Non-canonical D-amino Acids. EMBO J..

[59] Lam H., Oh D.-C., Cava F., Takacs C.N., Clardy J., De Pedro M.A., Waldor M.K. (2009). D-Amino Acids Govern Stationary Phase Cell Wall Remodeling in Bacteria. Science.

[60] Hernández S.B., Dörr T., Waldor M.K., Cava F. (2020). Modulation of Peptidoglycan Synthesis by Recycled Cell Wall Tetrapeptides. Cell Rep..

[61] Wild J., Hennig J., Lobocka M., Walczak W., Klopotowski T. (1985). Identification of the dadX Gene Coding for the Predominant Isozyme of Alanine Racemase in *Escherichia coli* K12. Mol. Gen. Genet..

[62] Hernández S.B., Castanheira S., Pucciarelli M.G., Cestero J.J., Rico-Pérez G., Paradela A., Ayala J.A., Velázquez S., San-Félix A., Cava F. (2022). Peptidoglycan Editing in Non-Proliferating Intracellular Salmonella as Source of Interference with Immune Signaling. PLoS Pathog..

[63] Signoretto C., Lleò M.D.M., Canepari P. (2002). Modification of the Peptidoglycan of *Escherichia coli* in the Viable But Nonculturable State. Curr. Microbiol..

[64] Paradis-Bleau C., Markovski M., Uehara T., Lupoli T.J., Walker S., Kahne D.E., Bernhardt T.G. (2010). Lipoprotein Cofactors Located in the Outer Membrane Activate Bacterial Cell Wall Polymerases. Cell.

[65] Sardis M.F., Bohrhunter J.L., Greene N.G., Bernhardt T.G. (2021). The LpoA Activator Is Required to Stimulate the Peptidoglycan Polymerase Activity of Its Cognate Cell Wall Synthase PBP1a. Proc. Natl. Acad. Sci. USA.

[66] Typas A., Banzhaf M., Van Den Berg Van Saparoea B., Verheul J., Biboy J., Nichols R.J., Zietek M., Beilharz K., Kannenberg K., Von Rechenberg M. (2010). Regulation of Peptidoglycan Synthesis by Outer-Membrane Proteins. Cell.

[67] Kermani A.A., Biboy J., Vollmer D., Vollmer W. (2022). Outer Membrane-Anchoring Enables LpoB to Regulate Peptidoglycan Synthesis Rate. Cell Surf..

[68] Shlosman I., Vettiger A., Bernhardt T.G., Kruse A.C., Loparo J.J. (2025). The Hit-and-Run of Cell Wall Synthesis: LpoB Transiently Binds and Activates PBP1b through a Conserved Allosteric Switch. Nat. Commun..

[69] Deghelt M., Cho S.-H., Sun J., Govers S.K., Janssens A., Dachsbeck A.V., Remaut H.K., Huang K.C., Collet J.-F. (2025). Peptidoglycan–Outer Membrane Attachment Generates Periplasmic Pressure to Prevent Lysis in Gram-Negative Bacteria. Nat. Microbiol..

[70] Rojas E.R., Billings G., Odermatt P.D., Auer G.K., Zhu L., Miguel A., Chang F., Weibel D.B., Theriot J.A., Huang K.C. (2018). The Outer Membrane Is an Essential Load-Bearing Element in Gram-Negative Bacteria. Nature.

[71] Son J.E., Park S.H., Choi U., Lee C.-R. (2024). Lytic Transglycosylase Repertoire Diversity Enables Intrinsic Antibiotic Resistance and Daughter Cell Separation in *Escherichia coli* under Acidic Stress. Antimicrob. Agents Chemother..

[72] Choi U., Park S.H., Lee H.B., Son J.E., Lee C.-R. (2023). Coordinated and Distinct Roles of Peptidoglycan Carboxypeptidases DacC and DacA in Cell Growth and Shape Maintenance under Stress Conditions. Microbiol. Spectr..

[73] Nallamotu K.C., Bahadur R., Kaul M., Reddy M. (2023). Peptidoglycan Remodeling by an L,D-Transpeptidase, LdtD during Cold Shock in *Escherichia coli*. J. Bacteriol..

[74] Irving S.E., Choudhury N.R., Corrigan R.M. (2021). The Stringent Response and Physiological Roles of (Pp)pGpp in Bacteria. Nat. Rev. Microbiol..

[75] Rodionov D.G., Ishiguro E.E. (1995). Direct Correlation between Overproduction of Guanosine 3′,5′-Bispyrophosphate (ppGpp) and Penicillin Tolerance in *Escherichia coli*. J. Bacteriol..

[76] Ishiguro E.E., Ramey W.D. (1980). Inhibition of *in Vitro* Peptidoglycan Biosynthesis in *Escherichia coli* by Guanosine 5′-Diphosphate 3′-Diphosphate. Can. J. Microbiol..

[77] Roghanian M., Semsey S., Løbner-Olesen A., Jalalvand F. (2019). (P)ppGpp-Mediated Stress Response Induced by Defects in Outer Membrane Biogenesis and ATP Production Promotes Survival in *Escherichia coli*. Sci. Rep..

[78] Büke F., Grilli J., Cosentino Lagomarsino M., Bokinsky G., Tans S.J. (2022). ppGpp Is a Bacterial Cell Size Regulator. Curr. Biol..

[79] Alvarez L., Hernandez S.B., Torrens G., Weaver A.I., Dörr T., Cava F. (2024). Control of Bacterial Cell Wall Autolysins by Peptidoglycan Crosslinking Mode. Nat. Commun..

[80] Giacomucci S., Alvarez L., Rodrigues C.D.A., Cava F., Paradis-Bleau C. (2022). Hydroxyl Radical Overproduction in the Envelope: An Achilles’ Heel in Peptidoglycan Synthesis. Microbiol. Spectr..

[81] Wang G., Olczak A., Forsberg L.S., Maier R.J. (2009). Oxidative Stress-Induced Peptidoglycan Deacetylase in Helicobacter Pylori. J. Biol. Chem..

[82] Peters K., Pazos M., Edoo Z., Hugonnet J.-E., Martorana A.M., Polissi A., VanNieuwenhze M.S., Arthur M., Vollmer W. (2018). Copper Inhibits Peptidoglycan LD-Transpeptidases Suppressing β-Lactam Resistance Due to Bypass of Penicillin-Binding Proteins. Proc. Natl. Acad. Sci. USA.

[83] Sperandeo P., Villa R., Martorana A.M., Šamalikova M., Grandori R., Dehò G., Polissi A. (2011). New Insights into the Lpt Machinery for Lipopolysaccharide Transport to the Cell Surface: LptA-LptC Interaction and LptA Stability as Sensors of a Properly Assembled Transenvelope Complex. J. Bacteriol..

[84] Sperandeo P., Lau F.K., Carpentieri A., De Castro C., Molinaro A., Dehò G., Silhavy T.J., Polissi A. (2008). Functional Analysis of the Protein Machinery Required for Transport of Lipopolysaccharide to the Outer Membrane of *Escherichia coli*. J. Bacteriol..

[85] Martorana A.M., Motta S., Di Silvestre D., Falchi F., Dehò G., Mauri P., Sperandeo P., Polissi A. (2014). Dissecting *Escherichia coli* Outer Membrane Biogenesis Using Differential Proteomics. PLoS ONE.

[86] Gurnani Serrano C.K., Winkle M., Martorana A.M., Biboy J., Morè N., Moynihan P., Banzhaf M., Vollmer W., Polissi A. (2021). ActS Activates Peptidoglycan Amidases during Outer Membrane Stress in *Escherichia coli*. Mol. Microbiol..

[87] Mueller E.A., Iken A.G., Ali Öztürk M., Winkle M., Schmitz M., Vollmer W., Di Ventura B., Levin P.A. (2021). The Active Repertoire of *Escherichia coli* Peptidoglycan Amidases Varies with Physiochemical Environment. Mol. Microbiol..

[88] Moffatt J.H., Harper M., Harrison P., Hale J.D.F., Vinogradov E., Seemann T., Henry R., Crane B., Michael F.S., Cox A.D. (2010). colistin Resistance in *Acinetobacter baumannii* Is Mediated by Complete Loss of Lipopolysaccharide Production. Antimicrob. Agents Chemother..

[89] Simpson B.W., Nieckarz M., Pinedo V., McLean A.B., Cava F., Trent M.S. (2021). *Acinetobacter baumannii* Can Survive with an Outer Membrane Lacking Lipooligosaccharide Due to Structural Support from Elongasome Peptidoglycan Synthesis. mBio.

[90] Kang K.N., Kazi M.I., Biboy J., Gray J., Bovermann H., Ausman J., Boutte C.C., Vollmer W., Boll J.M. (2021). Septal Class A Penicillin-Binding Protein Activity and LD-Transpeptidases Mediate Selection of colistin-Resistant Lipooligosaccharide-Deficient *Acinetobacter baumannii*. mBio.

[91] Nandy S., Tehrani A.F., Hunt-Serracin A.C., Biboy J., Pybus C., Vollmer W., Boll J.M. (2025). Molecular Interplay between Peptidoglycan Integrity and Outer Membrane Asymmetry in Maintaining Cell Envelope Homeostasis. J. Bacteriol..

[92] Malinverni J.C., Silhavy T.J. (2009). An ABC Transport System That Maintains Lipid Asymmetry in the Gram-Negative Outer Membrane. Proc. Natl. Acad. Sci. USA.

[93] Olea-Ozuna R.J., Furlan B., Gong H., Massidda O., Boll J.M. (2026). Outer Membrane Remodeling via Lipid-Peptidoglycan Crosstalk Enables Lipooligosaccharide-Deficient colistin Resistance. Cell Rep..

[94] Kitano K., Tuomanen E., Tomasz A. (1986). Transglycosylase and Endopeptidase Participate in the Degradation of Murein during Autolysis of *Escherichia coli*. J. Bacteriol..

[95] Tipper D.J. (1985). Mode of Action of β-Lactam Antibiotics. Pharmacol. Ther..

[96] Barna J.C.J., Williams D.H. (1984). The Structure and Mode of Action of Glycopeptide Antibiotics of the Vancomycin Group. Annu. Rev. Microbiol..

[97] Satta G., Cornaglia G., Mazzariol A., Golini G., Valisena S., Fontana R. (1995). Target for Bacteriostatic and Bactericidal Activities of Beta-Lactam Antibiotics against *Escherichia coli* Resides in Different Penicillin-Binding Proteins. Antimicrob. Agents Chemother..

[98] Heidrich C., Templin M.F., Ursinus A., Merdanovic M., Berger J., Schwarz H., De Pedro M.A., Höltje J. (2001). Involvement of *N*-acetylmuramyl-L-alanine Amidases in Cell Separation and Antibiotic-induced Autolysis of *Escherichia coli*. Mol. Microbiol..

[99] Kohlrausch U., Höltje J.V. (1991). Analysis of Murein and Murein Precursors during Antibiotic-Induced Lysis of *Escherichia coli*. J. Bacteriol..

[100] Cho H., Uehara T., Bernhardt T.G. (2014). Beta-Lactam Antibiotics Induce a Lethal Malfunctioning of the Bacterial Cell Wall Synthesis Machinery. Cell.

[101] Dörr T. (2021). Understanding Tolerance to Cell Wall–Active Antibiotics. Ann. N. Y. Acad. Sci..

[102] Dörr T., Davis B.M., Waldor M.K. (2015). Endopeptidase-Mediated Beta Lactam Tolerance. PLoS Pathog..

[103] Dörr T., Alvarez L., Delgado F., Davis B.M., Cava F., Waldor M.K. (2016). A Cell Wall Damage Response Mediated by a Sensor Kinase/Response Regulator Pair Enables Beta-Lactam Tolerance. Proc. Natl. Acad. Sci. USA.

[104] Shin J.-H., Choe D., Ransegnola B., Hong H.-R., Onyekwere I., Cross T., Shi Q., Cho B.-K., Westblade L.F., Brito I.L. (2021). A Multifaceted Cellular Damage Repair and Prevention Pathway Promotes High-Level Tolerance to β-Lactam Antibiotics. EMBO Rep..

[105] Narzary J., Mohiuddin S.G., Massahi A., Orman M.A. (2026). Tail-Specific Protease (Tsp)-Mediated Envelope Remodeling and Beta-Lactam Tolerance in *Escherichia coli*. Microbiol. Spectr..

[106] Weaver A.I., Murphy S.G., Umans B.D., Tallavajhala S., Onyekwere I., Wittels S., Shin J.-H., VanNieuwenhze M., Waldor M.K., Dörr T. (2018). Genetic Determinants of Penicillin Tolerance in Vibrio Cholerae. Antimicrob. Agents Chemother..

[107] Thomson N.M., Turner A.K., Yasir M., Bastkowski S., Lott M., Webber M.A., Charles I.G. (2022). A Whole-Genome Assay Identifies Four Principal Gene Functions That Confer Tolerance of Meropenem Stress upon *Escherichia coli*. Front. Antibiot..

[108] Held K., Gasper J., Morgan S., Siehnel R., Singh P., Manoil C. (2018). Determinants of Extreme β-Lactam Tolerance in the Burkholderia Pseudomallei Complex. Antimicrob. Agents Chemother..

[109] Park S.H., Choi U., Ryu S.-H., Lee H.B., Lee J.-W., Lee C.-R. (2022). Divergent Effects of Peptidoglycan Carboxypeptidase DacA on Intrinsic β-Lactam and Vancomycin Resistance. Microbiol. Spectr..

[110] Kimura T., Ishikawa K., Nakagawa R., Furuta K., Kaito C. (2025). Lytic Transglycosylase Deficiency Increases Susceptibility to β-lactam Antibiotics But Reduces Susceptibility to Vancomycin in *Escherichia coli*. Microbiol. Immunol..

[111] El-Araby A.M., Fisher J.F., Mobashery S. (2025). Bacterial Peptidoglycan as a Living Polymer. Curr. Opin. Chem. Biol..

[112] Melotti E., Delerue T., Gomperts Boneca I. (2025). Understanding How Bacterial Cell Wall Peptidoglycan Metabolism Can Be Used to Develop Antimicrobial Strategies. Expert Opin. Ther. Targets.

[113] Martinez-Bond E.A., Soriano B.M., Williams A.H. (2022). The Mechanistic Landscape of Lytic Transglycosylase as Targets for Antibacterial Therapy. Curr. Opin. Struct. Biol..

[114] Weaver A.I., Alvarez L., Rosch K.M., Ahmed A., Wang G.S., Van Nieuwenhze M.S., Cava F., Dörr T. (2022). Lytic Transglycosylases Mitigate Periplasmic Crowding by Degrading Soluble Cell Wall Turnover Products. eLife.

[115] Bonis M., Williams A., Guadagnini S., Werts C., Boneca I.G. (2012). The Effect of Bulgecin A on Peptidoglycan Metabolism and Physiology of *Helicobacter pylori*. Microb. Drug Resist..

[116] Williams A., Wheeler R., Thiriau C., Haouz A., Taha M., Boneca I. (2017). Bulgecin A: The Key to a Broad-Spectrum Inhibitor That Targets Lytic Transglycosylases. Antibiotics.

[117] Skalweit M.J., Li M. (2016). Bulgecin A as a β-Lactam Enhancer for Carbapenem-Resistant *Pseudomonas aeruginosa* and Carbapenem-Resistant *Acinetobacter baumannii* Clinical Isolates Containing Various Resistance Mechanisms. Drug Des. Dev. Ther..

[118] Tomoshige S., Dik D.A., Akabane-Nakata M., Madukoma C.S., Fisher J.F., Shrout J.D., Mobashery S. (2018). Total Syntheses of Bulgecins A, B, and C and Their Bactericidal Potentiation of the β-Lactam Antibiotics. ACS Infect. Dis..

[119] Kim C., Tomoshige S., Lee M., Zgurskaya H.I., Mobashery S. (2023). Penetration through Outer Membrane and Efflux Potential in Pseudomonas Aeruginosa of Bulgecin A as an Adjuvant to β-Lactam Antibiotics. Antibiotics.

[120] Liu Y., Frirdich E., Taylor J.A., Chan A.C.K., Blair K.M., Vermeulen J., Ha R., Murphy M.E.P., Salama N.R., Gaynor E.C. (2016). A Bacterial Cell Shape-Determining Inhibitor. ACS Chem. Biol..

[121] Ho C.S., Wong C.T.H., Aung T.T., Lakshminarayanan R., Mehta J.S., Rauz S., McNally A., Kintses B., Peacock S.J., De La Fuente-Nunez C. (2025). Antimicrobial Resistance: A Concise Update. Lancet Microbe.

[122] Crysler A., De La Fuente-Nunez C. (2026). Mining the Code of Life for New Antibiotics. Cell Host Microbe.

[123] Culp E.J., Waglechner N., Wang W., Fiebig-Comyn A.A., Hsu Y.-P., Koteva K., Sychantha D., Coombes B.K., Van Nieuwenhze M.S., Brun Y.V. (2020). Evolution-Guided Discovery of Antibiotics That Inhibit Peptidoglycan Remodelling. Nature.

